# Improving carbon modeling in aspen‐dominated forests: Lessons from CBM‐CFS3 evaluation against long‐term field data

**DOI:** 10.1002/eap.70307

**Published:** 2026-09-18

**Authors:** Nicole Lau, Derek Sattler, Michael Michaelian, Oleksandra Hararuk

**Affiliations:** ^1^ Natural Resources Canada Canadian Forest Service, Northern Forestry Centre Edmonton Alberta Canada; ^2^ Natural Resources Canada Canadian Forest Service, Pacific Forestry Centre Victoria British Columbia Canada

**Keywords:** boreal forests, carbon modeling, CBM‐CFS3, forest carbon cycling, *Populus tremuloides*, trembling aspen

## Abstract

Boreal forests cover a substantial portion of Canada's land base and play a central role in the national carbon budget through carbon storage and fluxes across multiple above‐ and belowground pools. The Carbon Budget Model of the Canadian Forest Sector (CBM‐CFS3) simulates and projects forest carbon dynamics under varying conditions and management scenarios, and ongoing model evaluation and calibration are essential to maintain accuracy in carbon monitoring, accounting, and reporting. This study assesses CBM‐CFS3 performance using independent ground plot data from 64 aspen‐dominated stands that have been monitored since 2000 as part of the Climate Impacts on the Productivity and Health of Aspen (CIPHA) project. We compared modeled and observed C stocks in aboveground biomass, snags, forest floor, and mineral soil pools, and examined the influence of stand characteristics, climate variables, and soil chemical properties on model residuals. Model performance varied across carbon pools: CBM‐CFS3 most accurately simulated aboveground biomass C stocks, performed moderately well for snags, but failed to capture spatial variability in forest floor and mineral soil C stocks. Our findings suggest that incorporating stand density into the yield curve model and stand age into volume‐to‐biomass conversion equations would enhance the accuracy of aboveground biomass C stock estimates in aspen‐dominated stands. Representation of snag carbon dynamics could be improved by accounting for lagged mortality following drought events and by increasing snag fall rates. Further refinement of forest floor carbon dynamics will require integrating the influence of moisture on organic matter decomposition, while improving mineral soil carbon dynamics will require incorporating the effect of clay content on decomposition and reducing the temperature sensitivity of decay. Because these pools are interdependent, refinements in one pool will have cascading effects on downstream pools and overall carbon dynamics. The CBM‐CFS3 remains a key tool for national carbon estimation, and the availability of high spatial and temporal resolution data, such as CIPHA, for model evaluation will further strengthen the model's predictive capability.

## INTRODUCTION

Boreal forests play an important role in the global carbon (C) cycle, accounting for 20% of the global forest C uptake and 32% of the global forest C storage (Pan et al., 2011). In Canada, boreal forests cover approximately 38% of the land area (Natural Resources Canada, 2020) and therefore represent a central component of the national C budget. Forest C stocks are distributed among living and dead biomass, litter, and soil organic carbon (SOC) pools. Soils hold most of the C in Canada's forests, containing an estimated 52.6–66.1 Gt C (Bradshaw & Warkentin, 2015), while 18.3 Gt C is stored in the living biomass (77% of which is in aboveground biomass) and 2.6 Gt C in litter (Sothe et al., 2022). Carbon dynamics within these pools are driven by vegetation growth, mortality, and decomposition, which together influence the net carbon balance of forest stands.

Trembling aspen (*Populus tremuloides*) is the most abundant broadleaf tree species in Canada and has a transcontinental distribution. Forests with leading species of the *Populus* genus account for roughly 10% of Canada's productive forest area and 11% of its aboveground biomass (Power & Gillis, 2006). Specifically, trembling aspen–dominated forests make up a quarter of the country's broadleaf forest area (Power & Gillis, 2006). Given their widespread distribution, it is important to accurately quantify C stocks and fluxes in aspen‐dominated ecosystems to fully capture their contributions to national carbon accounting.

Improving estimates of forest C stocks and fluxes supports Canada's reporting commitments under international climate agreements. Canada's National Forest Carbon Monitoring, Accounting, and Reporting System (NFCMARS; Kurz & Apps, 2006) provides annual estimates of C stocks and greenhouse gas sources and sinks within managed forests. This system fulfills reporting obligations under the United Nations Framework Convention on Climate Change (UNFCCC). Methodologies used in this system comply with international standards outlined by the Intergovernmental Panel on Climate Change (IPCC) Good Practice Guidance for Land Use, Land‐use Change and Forestry (Penman et al., 2003), and Guidelines for National Greenhouse Gas Inventories (Eggleston et al., 2006).

While site‐level C stocks and fluxes can be estimated through ground‐based measurements, scaling these measurements up to regional or national levels, and over multiple years, requires process‐based models. In Canada, the Carbon Budget Model of the Canadian Forest Sector (CBM‐CFS3; Kurz et al., 2009) serves as the primary modeling framework used to estimate C stocks and fluxes in managed forests. The CBM‐CFS3 is an inventory‐based model that simulates above‐ and belowground forest C dynamics by integrating stand‐specific forest inventory data with ecological parameters from national databases. The model estimates C stocks and fluxes through various live and dead pools over time as trees grow, die, and decompose. CBM‐CFS3 is integrated with the NFCMARS to inform forest carbon dynamics under different disturbance and management scenarios, with applications at various scales within Canada (e.g. Amichev et al., 2016; Chen et al., 2016; Dymond et al., 2016; Kurz et al., 2008), and internationally (e.g. Cienciala & Melichar, 2024; Olguin et al., 2018; Pilli et al., 2017; Tang et al., 2022).

Ongoing validation and calibration of the CBM‐CFS3 are essential to produce reliable estimates of forest C stocks and fluxes. Previous comparisons of model outputs with field data have led to important model improvements. Using National Forest Inventory ground plots, Shaw et al. (2014) confirmed that the model reliably simulated aboveground biomass but found major uncertainties in total ecosystem C stock estimates, largely due to errors in simulated SOC and deadwood C. Hararuk et al. (2017) used the same dataset to calibrate CBM‐CFS3 parameters regulating litterfall and dead organic matter decay, which reduced model bias in simulating deadwood and soil C pools. The authors also demonstrated the distinct decay patterns in woody debris originating from hardwood and softwood tree species as well as greater temperature sensitivity of recalcitrant soil C pools than originally assumed in the model. In addition, Shaw et al. (2015) analyzed 2391 soil profiles and showed that including species‐ and soil type‐specific decay parameters improved simulation of soil C dynamics. Bernier et al.'s (Bernier et al., 2010) work in eastern Canadian boreal stands identified yield curve errors, particularly in disturbed or data‐scarce landscapes, as the main source of model inaccuracies, further exacerbated by inconsistent growth and yield modeling practices across jurisdictions. Because comprehensive evaluations using independent ground data are limited, largely due to the scarcity of suitable validation datasets, these studies provide valuable insights for advancing CBM‐CFS3 performance.

Building on this foundation, the current study evaluates model performance over a longer timeframe by leveraging data from the Climate Impacts on Productivity and Health of Aspen (CIPHA) study, a long‐term monitoring network. This dataset features a consistent sampling design, well‐documented site histories, and reliable estimates of aboveground C dynamics over time. The CIPHA study was initiated by the Canadian government in response to public concern over widespread trembling aspen dieback, with preliminary work beginning in the late 1990s (Hogg et al., 2008). The first CIPHA site was established in 2000, and the network has since provided continuous site data across broad environmental gradients (Hogg et al., 2008). The CIPHA study tracks aspen forest health in regions vulnerable to severe drought and includes comprehensive information to assess biomass dynamics, mortality, carbon transfers to and from standing deadwood, and initial soil C stocks in the forest floor and upper mineral soil across trembling aspen–dominated stands in multiple ecozones.

The objectives of the current study were to (1) evaluate the performance of the CBM‐CFS3 by comparing modeled C stock predictions against field measured data from CIPHA in (a) aboveground biomass, (b) snags, (c) forest floor, (d) mineral soil; and to (2) identify model components that could be refined to improve simulation of carbon dynamics in these pools. Improving model performance will enable more accurate carbon accounting in Canada's aspen forests.

## METHODS

### Observed data

**FIGURE 1:**
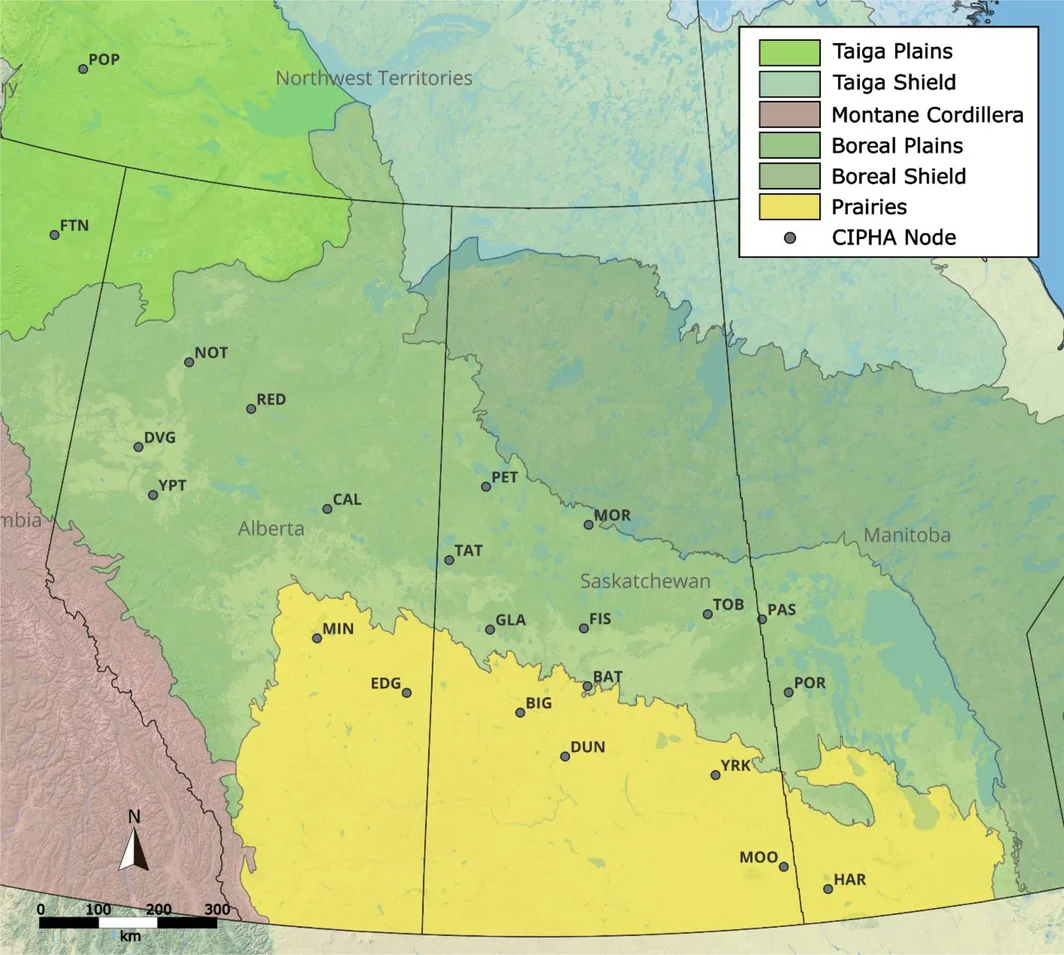
Maps of study locations from stands in the Climate Impacts on the Productivity and Health of Aspen (CIPHA) project. Black points indicate the subset of 64 CIPHA monitoring stands included in this study located in the boreal forest and parkland regions of Canada. Background colors represent ecozones based on the Ecological Framework of Canada. The stands were established in 2000 with ongoing remeasurements to track the effects of climate change on aspen health and dieback over time.

Field data were sourced from the CIPHA study, which established a network of 30 long‐term monitoring study sites (“nodes”) across Canada in 2000 (Figure 1). Each node contained three stands located within 25 km of one another, for a total of 90 stands. Stands were set up in unmanaged, pure trembling aspen (*Populus tremuloides* Michx.) forests (≥80% aspen) without human intervention, in boreal forests and aspen parklands. Within each stand, two plots were established no more than 100 m apart. For meaningful model evaluation, we selected a subset of stands (*n* = 64) located in British Columbia, Alberta, Saskatchewan, Manitoba, and the Northwest Territories. Stands lacking consistent or complete ground measurements across all survey years were excluded. Specifically, this applied to stands missing survey years, such as those in Ontario (*n* = 12), and those destroyed by major disturbances such as severe wildfire, blowdown, or logging (*n* = 9). Additionally, stands without site index information necessary for yield curve generation were removed (*n* = 5). Elevations of the retained stands ranged from 204 to 784 m. Stands were distributed along a temperature and moisture gradient. Historical (1901–2024) mean annual temperature (MAT) ranged between −7.6 and 6.3°C among the plots, mean annual precipitation (MAP) varied from 18.0 to 85.9 mm per year, and annual climate moisture index (CMI), defined as the difference between MAP and potential evapotranspiration (PET), ranged from −47.2 to 41.5 mm (Régnière et al., 2017). Soils characteristic of these regions included Gray Luvisols in the boreal and Black or Dark Brown Chernozems in parkland regions (Clayton et al., 1977; Hogg et al., 2008).

The CIPHA stands were approximately 40 to 80 years old when monitoring started. Stand age for each plot was determined using tree ring analyses of basal disks collected in 2000 (Hogg et al., 2008). Selected stands were continuous forests of at least 250 m by 150 m in size, although the minimum was sometimes reduced to 100 m by 100 m in parkland areas where stands were small, clonal, and surrounded by grasslands. All stands were required to be free from significant disturbance or dieback prior to the onset of monitoring and situated at least 1 km from point‐source pollution and waterbodies. Within each stand, two rectangular plots, 10 m wide by approximately 15–35 m long, were established to capture at least 25 live aspen trees at the time of plot establishment. Where feasible, plots were located so that all plot boundaries were at least 50 m from the forest edge. All trees (living and dead) with dbh >7 cm were labeled and numbered. Tree health data (species, dominance, percent crown dieback, live/dead status, health status) were recorded annually for most stands, and tree mensuration (dbh, height, recruitment) was conducted every 4 years. For the subset of trees for which heights were not measured in the field, they were estimated using height‐diameter models developed for major Alberta tree species that incorporated subregional parameters for Alberta nodes or generalized provincial‐level parameters for non‐Alberta nodes (S. Huang, Yang, & Aitkin, 2013). Soil samples (*n* = 1–3 per plot) were collected in 2002 from the forest floor and the top 10 cm of mineral soil for analysis of bulk density, texture, pH, electrical conductivity, cation exchange capacity, and chemistry. The variables measured and their sampling frequency are summarized in Table 1. See Hogg et al. (2008) for full study design and sampling details.

**TABLE 1:** Variables measured in the Climate Impacts on Productivity and Health of Aspen (CIPHA) long‐term monitoring stands.

Data type	Variables measured	Measurement frequency
Tree health and status	Species, dominance, crown dieback (%), tree status (live/dead), pest and disease incidence/severity	Varies by node^a^
Tree mensuration	Top height, diameter at breast height (dbh)	Every 4 years; from 2000 onwards
Soil: Forest floor	Bulk density (*n* = 3 per plot): LFH depth (at four corners of a 15 × 15 cm fixed area quadrat), sample volume Chemical analyses (*n* = 1 per plot, except for nodes FIS and BAT where *n* = 3 per plot): pH, electrical conductivity (EC), cation exchange capacity (CEC), chemical composition	Measured once; 2002
Soil: Mineral soil	Bulk density (*n* = 3 per plot): to a depth of 10 cm using a 10 cm diameter slide‐hammer soil corer Chemical analyses (*n* = 1 per plot, except for nodes FIS and BAT where *n* = 2 to 3 per plot) from the top 15 cm of the mineral soil: texture, pH, EC, CEC, chemical composition	Measured once; 2002
Site productivity	Site index	Measured once; 2000

*Note*: List of the variables relevant to this study and their measurement frequency across the subset of 64 CIPHA monitoring stands included in this study (2 plots per stand) in boreal forests and parkland regions of Canada. The stands were established in 2000 with ongoing remeasurements to track the effects of climate change on aspen health and dieback over time. LFH, litter, fermenting, humified ‐ the states of organic matter in the forest floor.

^a^ Tree health and status variables measured annually from 2000 (CAL, DVG, EDG, MIN, NOT, RED, YPT); annually 2000–2008 then every 4 years from 2012 (FTN, HAR, MOO, MOR, PAS, PET, POP, POR, TOB, YRK); or annually 2000–2012 then every 4 years from 2016 (BAT, BIG, DUN, FIS, GLA, TAT).

Carbon stocks were calculated for multiple biomass and dead organic matter pools using field data. Aboveground biomass for individual trees was estimated using species‐specific Canadian national allometric equations based on dbh and height (Ung et al., 2008). We summed aboveground biomass within each plot and calculated the stand‐level biomass by taking plot‐area‐weighted averages and scaling to per‐hectare values. Aboveground biomass was converted to C stocks by applying a factor of 0.5, based on the assumption that dry biomass contains 50% carbon. We estimated biomass for individual snags using the same Ung et al. (2008) allometric equations as an initial proxy. Since decay class information was not available, we adjusted these estimates to account for expected differences between live trees and snags of equivalent dbh. Biomass components were reduced according to Smith et al. (2003) to broadly approximate biomass loss from structural damage and decay. Specifically, stemwood and bark were each reduced by 10%, branch biomass by 33%, and foliage biomass by 100%. Forest floor SOC stocks for each sample were calculated as the product of bulk density, forest floor thickness, and C content of the sample. If multiple samples were available within a plot, we averaged the forest floor C stocks calculated for each sample. To calculate stand‐level forest floor SOC (tonnes per hectare), we took a plot‐area‐weighted average of the two plot‐level SOC estimates. Mineral soil C stocks were calculated as the product of the sample's bulk density, its carbon content, and a fixed sampling depth of 10 cm. We then averaged the sample‐level mineral SOC stocks to get the plot‐level mineral SOC estimates and scaled them to stand level using plot‐area‐weighted averages, similar to the forest floor SOC calculations.

We represented mortality and snag fall rates as carbon transfers between forest pools. Annual mortality was calculated as the C stocks of trees that became snags divided by the total live aboveground biomass in the current survey year, representing the proportion of live tree carbon that transitioned into the snag pool by the subsequent survey. Snag fall rate was calculated as the C stocks of snags that fell divided by the total snag carbon in the current survey year, representing the proportion of snag carbon that moved into the downed log pool by the subsequent survey. Both rates were normalized by the 4‐year time interval between remeasurements. As downed trees were not directly measured in the CIPHA study, we estimated C stocks of fallen snags using their measurements from the most recent survey year.

Site index was calculated for each stand using dendrochronological disks from aspen trees collected in 2000. To minimize plot‐level growth bias, one tree from each of three diameter size classes, small, medium, and large, each representing one‐third of the total basal area, was felled and used for site index calculation. Site index was then calculated according to Huang's (1997) model using provincial aspen parameters.

### CBM‐CFS3 simulations

We simulated C dynamics at each CIPHA stand to assess the performance of the CBM‐CFS3 in representing observed estimates of aboveground biomass, standing snags, tree mortality, snag fall rates, and SOC stocks in forest floor and top mineral soil. From the original 90 stands, 64 met the inclusion criteria and were used in the current study. Model simulations were conducted using CBM‐CFS3 (v1.2.8956.389), with standard import files created for each stand. Default parameters related to carbon turnover and decay, including dead organic matter and biomass turnover rates, were modified based on values calibrated against National Forest Inventory ground plot data to address known model biases (Hararuk et al., 2017).

#### Model inputs

Carbon dynamics in CBM‐CFS3 are driven by empirical yield curves (dynamics of merchantable wood volume over stand age), mean annual temperature (MAT), and disturbance history. In addition, information on stand age at the time of measurement is needed to match the simulated C stocks to the observed estimates. To generate the yield curves for CIPHA stands, a nonlinear mixed‐effects volume‐age model was developed and fitted using a modified version of the volume‐age equation from (Huang et al., 2009) (Equation 1):

(1) $${\mathrm{Vol}}_{i}=b_{1}+u_{1i}\times {\mathrm{Age}}^{b_{2}+u_{2i}}\times \exp\left[-\left(b_{1}+u_{1i}\right)\times {\mathrm{Age}}_{i}\right]+\epsilon_{i}$$

where ${\mathrm{Vol}}_{i}$ is gross merchantable volume for the *i*th stand, in cubic meters per hectare; Age is stand age in years; $b_{1}$ and $b_{2}$ are fixed‐effect parameters; $u_{1i}$ and $u_{2i}$ are random effect parameters unique to the *i*th stand; and $\epsilon_{i}$ is the normally distributed within‐stand residual error. A first‐order autocorrelation structure was used to account for correlated errors in time. Model parameters were determined using maximum likelihood estimations (nlme R package v3.1.168; Pinheiro et al., 2025).

The calculation of observed gross merchantable volume for each stand began by first calculating individual tree volumes using total tree height (in meters) and dbh (in centimeters) (Posit team, 2024; J.‐G. Huang, Stadt, et al., 2013; Maltman et al., 2023). For plots in Alberta, Saskatchewan, and Manitoba, either the subregion‐specific (Alberta plots) or province‐wide (Saskatchewan and Manitoba plots) taper functions described in Huang (1994) were used, obtained from the CTAE R package (v0.4.3; Huang, 1994; Tompalski & Manvailer, 2025). For plots in British Columbia and the Northwest Territories, the Kozak 2002 variable‐exponent taper function was used (Kozak, 2004), implemented through the FAIBbase R package (v2.0.0; Kozak, 2004; Luo, 2025). For all plots, merchantable gross volume was calculated using a 13/7/30 utilization standard (minimum dbh of 13 cm, top diameter of 7 cm inside bark, stump height of 30 cm). Tree level volumes were aggregated to plot level volume and then area‐weighted across the two plots per stand to calculate the mean stand‐level gross merchantable volume.

Separate yield curves were developed for deciduous and coniferous components of each stand. For the deciduous component, an anchor point was added to the model fitting dataset at year 200 using a pre‐determined volume of 200 m^3^/ha, based on observations from some of the oldest aspen stands in Alberta's permanent sample plot databases. This prevented unrealistic over‐ or underprediction of aspen growth at ages beyond 140 years and produced gradually declining stand‐level volume at old ages, typical of aspen‐dominated stands. The aspen volume model had an RMSE of 26.44 m^3^/ha and an *R* ^2^ of 93%, while the coniferous model had an RMSE of 6.83 m^3^/ha and an *R* ^2^ of 39% (Appendix S1: Figure S2a,b). No systematic bias was observed when comparing predicted versus observed values.

MAT for model input was calculated as the mean of averages for annual maxima and minima temperatures derived from a national spatially interpolated climate database (McKenney et al., 2006) with data available from 1901 onward.

All stands were initialized with a stand‐replacing wildfire prior to being grown to their respective stand ages. Although all stands experienced some level of defoliation and background tree mortality since stand origin, a secondary disturbance was only applied when it caused at least a 10% reduction in aboveground biomass between survey years, which occurred in most stands (*n* = 44). All insect and defoliation events were simulated using a modified “Generic—95% mortality” disturbance type, which transfers 95% of hardwood biomass carbon to respective downstream dead organic matter (DOM) pools. In these events, the softwood component of the stands remained undisturbed. Disturbance events were scheduled to reflect the proportion of the stand's aboveground biomass lost between survey years according to the field data.

#### Simulation

In CBM‐CFS3, stands undergo a spin‐up process of repeated growth to the age of the fire return interval and fire events until the difference in slow soil carbon pools at the end of two successive rotations is less than 0.1% (Kull et al., 2019). The model assumes a default fire return interval of 125 years in the Boreal Plains and Taiga Plains, and 75 years in the Subhumid Prairies. Following the spin‐up phase, a final stand‐replacing wildfire event was applied, and the model was run forward for a 200‐year simulation period. In cases where field data indicated a secondary disturbance, such as foliar insects or woody tissue insects, the disturbances were scheduled in the model if they resulted in at least a 10% loss in aboveground biomass between survey years. We determined the percentage of aboveground biomass loss to represent the proportion of the stand to be disturbed. The affected portions of the targeted stands were reset to the beginning of the same growth curve after these disturbances, while the remaining unaffected portions were left to grow uninterrupted. Lastly, we extracted the simulated C stocks in aboveground biomass, standing snags, forest floor, and mineral soil at stand ages matching the observed data, and compared these values to the CIPHA estimates.

#### Comparison pools

CBM‐CFS3 simulates C dynamics in five living and nine DOM pools, whereas the CIPHA data facilitates calculating estimates of C stocks in one living pool (aboveground biomass), and three DOM pools (standing snags, forest floor, and mineral soil), which do not directly map to those in CBM‐CFS3. For instance, in CBM‐CFS3, merchantable wood volume from yield curves is converted to aboveground biomass components using the allometric equations and parameters from Boudewyn et al. (2007), with values specified according to species, ecozone, and province. Therefore, we summed the simulated C stocks in aboveground pools to make them comparable to CIPHA estimates. To generate simulated C stock estimates for the forest floor and mineral soil, we summed the simulated C stocks for the aboveground very fast and aboveground slow pools, and belowground very fast and belowground slow pools, respectively, following Shaw et al. (2014) and Hararuk et al. (2017). For snags, the simulated C stocks for the softwood stem snag, softwood branch snag, hardwood stem snag, and hardwood branch snag pools were summed.

CBM‐CFS3 simulates mineral SOC stocks for the whole soil profile, whereas CIPHA provides SOC estimates only for the top 10 cm of the mineral layer. To generate comparable simulated mineral SOC stocks, we scaled the whole profile SOC stocks to the top 10 cm. We used the Canadian upland forest soil profile and C stocks database (Shaw et al., 2018) to estimate parameters in a vertical cumulative C distribution equation following the logic of Jobbágy and Jackson (2000) (Equation 2):

(2) $$p=\frac{e^{a+b\times D}}{1+e^{a+b\times D}}$$

where p is the proportion of the mineral C stock in the top 100 cm of soil stored in the top D cm; a and b are estimated parameters. We assumed that p was beta‐distributed and used the R package “betareg” (Grün et al., 2012) to estimate a and b. Lastly, we subset soil profile data in Shaw et al. (2018) to only include profiles from trembling aspen–dominated stands located in Boreal Plains, Taiga Plains, or Subhumid Prairies. This ensured that estimated vertical soil C distribution pattern would be relevant for CIPHA plot locations. Parameter values and model fit are illustrated in Appendix S1: Figure S1 and Table S1.

### Evaluation of CBM‐CFS3 simulations

All statistical analyses were performed using RStudio (v2024.04.2; Posit team, 2024). We evaluated model performance using a combination of three metrics: root mean square error, average bias, and squared correlation coefficient between modeled estimates and observations, which represented the amount of spatiotemporal variation explained by the model. For the aboveground biomass and snag carbon pools, we included repeated field surveys conducted every 4 years over a 24‐year period. For the forest floor and mineral soil pools (0–10 cm depth), modeled versus observed SOC was assessed using data from a single sampling year in 2002. We used the adjusted coefficient of determination (*R* ^2^) to quantify the proportion of variance in the observed data explained by model predictions.

We also evaluated the dependency of CBM‐CFS3 residuals, defined as the difference between modeled and observed C stock estimates for each pool, on relevant climate, stand mensuration, and soil parameters by fitting mixed‐effects models using the lmerTest package (v3.1–3; Kuznetsova et al., 2017). For the forest floor and mineral soil carbon pools, where only one year of sampling was available, we averaged MAT, MAP, and CMI over the 20 years preceding sampling to buffer the influence of potential climate anomalies during the sample year (1982–2002). Model assumptions were visually assessed using the tab_model() function in the sjPlot package (v2.8.16; Lüdecke, 2025). We used the step() function in R to determine the best‐fitting model based on the lowest Akaike information criterion. For each final model, we assessed its adjusted *R* ^2^ and the *p*‐values associated with the sensitivities (slopes) of CBM‐CFS3 residuals to individual explanatory variables; slopes with *p*‐values <0.05 were considered statistically significant. Figures and summary tables for the marginal effects of significant predictors on CBM‐CFS3 residuals were generated using the sjPlot package (v2.8.16; Lüdecke, 2025).

## RESULTS

### Aboveground biomass carbon pool

**FIGURE 2:**
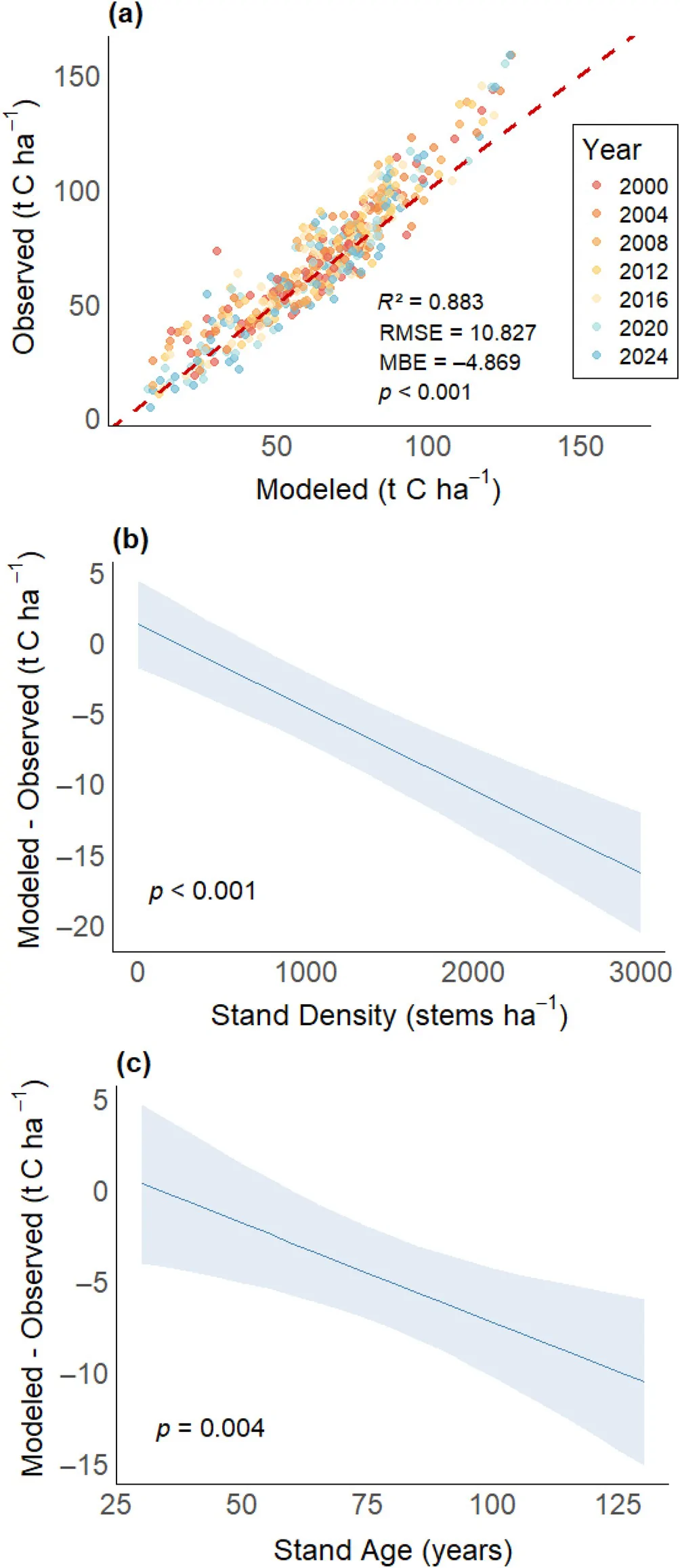
(a) Relationship between CBM‐CFS3 simulated and observed aboveground biomass carbon (C) stocks for 64 stands from the Climate Impacts on the Productivity and Health of Aspen (CIPHA) project. Points are colored by the year of field survey. The dashed 1:1 line denotes perfect agreement between modeled and observed values. A simple linear regression revealed a significant positive correlation (*p* < 0.001). Model performance evaluated using a combination squared correlation coefficient between modeled estimates and observations (*R* ^2^), root mean square error (RMSE), and mean bias error (MBE). Marginal effect of (b) stand density and (c) stand age were significant predictors of variation in CBM‐CFS3 residuals of the aboveground biomass C pool, derived from a linear mixed‐effects model.

Overall, the CBM‐CFS3 performed well in simulating aboveground biomass C stocks. Modeled and observed aboveground biomass C stocks were positively and significantly correlated (Figure 2a), with the CBM‐CFS3 explaining 88.3% of the variation in the observed data. On average, CBM‐CFS3 underestimated aboveground biomass C stocks by 4.87 t C/ha (Figure 2a). The lowest‐AIC model for the CBM‐CFS3 residuals was a linear mixed‐effects model with stand density and age set as fixed effects, along with node as a random effect. Node locations were widely distributed within Canada. Including node as a random effect accounted for spatial autocorrelation by reflecting that stands within the same node tended to be more similar to each other than to stands from different nodes. Stand density and age accounted for 7.4% of the variation in CBM‐CFS3 residuals, and when combined with the random effects of nodes, the model explained 45.6% of the variation. Significant negative relationships were found both between stand density and CBM‐CFS3 residuals (Figure 2b) and stand age and CBM‐CFS3 residuals (Figure 2c). CBM‐CFS3 tended to increasingly underestimate aboveground biomass C stocks with increasing stand density and age.

### Snag carbon pool

**FIGURE 3:**
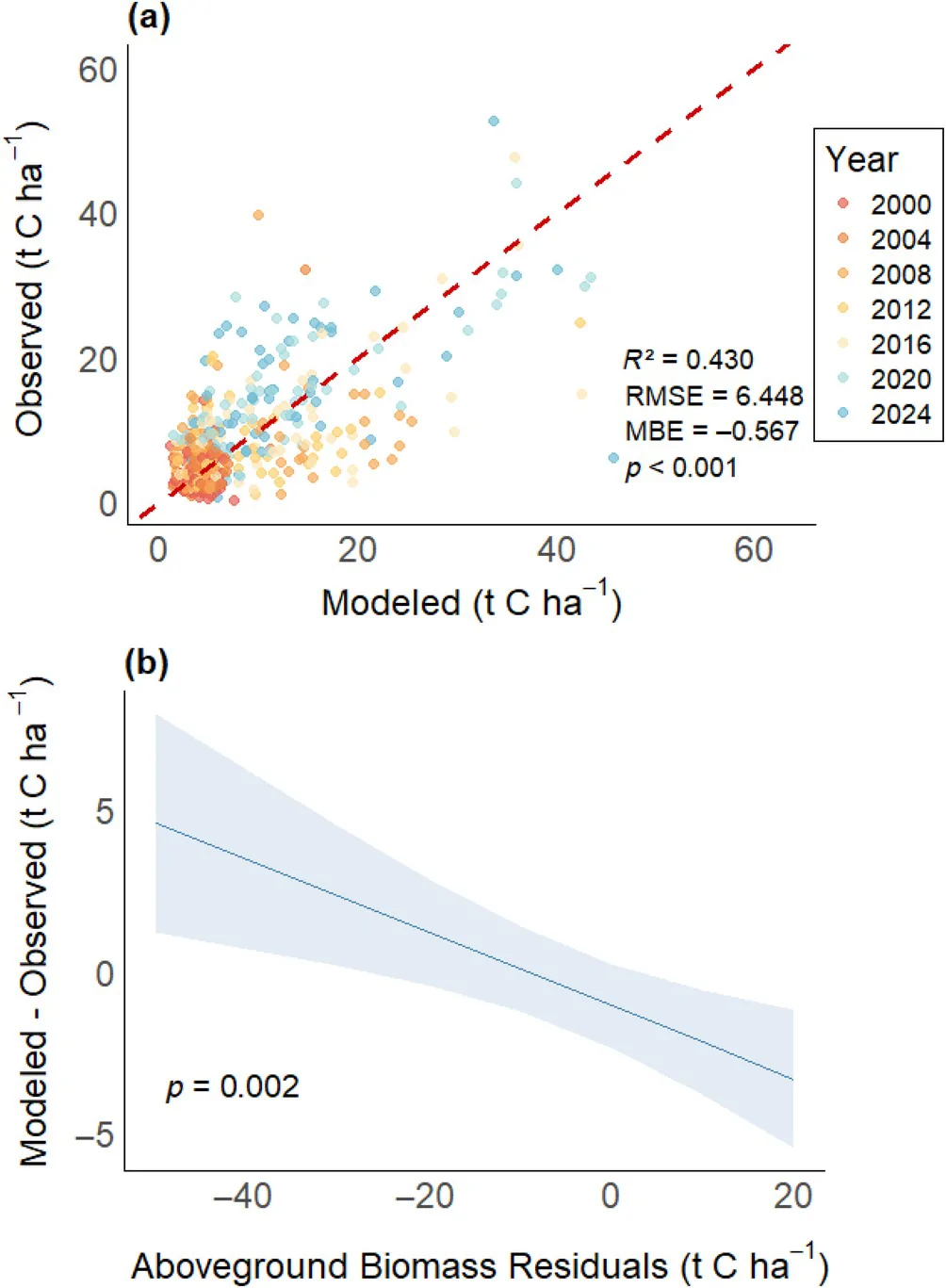
(a) Relationship between CBM‐CFS3 simulated and observed snag carbon (C) stocks for 64 stands from the Climate Impacts on the Productivity and Health of Aspen project. Points are colored by the year of field survey. The dashed 1:1 line denotes perfect agreement between modeled and observed values. A simple linear regression revealed a significant positive correlation (*p* < 0.001). Model performance evaluated using a combination squared correlation coefficient between modeled estimates and observations (*R* ^2^), root mean square error (RMSE), and mean bias error (MBE). (b) Marginal effect of aboveground biomass residuals, calculated as the difference between CBM‐CFS3 simulated and observed aboveground biomass C stocks, was a significant predictor of variation in CBM‐CFS3 residuals of the snag C pool, derived from a linear mixed‐effects model.

Simulated C stocks in snags were moderately and positively correlated with the observed estimates (Figure 3a), explaining approximately 43% of the variation in the observed data. On average, CBM‐CFS3 underestimated snag C stocks by 0.57 t C/ha. The best‐fitting model for the CBM‐CFS3 residuals in the snag C stocks was a linear mixed‐effects model with aboveground biomass residuals set as a fixed effect, and nodes as a random effect. Aboveground biomass residuals explained 2.8% of the variance in snag residuals, while the addition of the random effect of node increased the explained variance to 20.7%. Interestingly, stem snag C residuals were inversely related to the aboveground biomass residuals: CBM‐CFS3 tended to slightly overestimate stem snag C stocks at stands with underestimated aboveground biomass and slightly underestimate them in stands with overestimated aboveground biomass. Combining this pattern with findings in Figure 2b,c, snag C stocks tended to be increasingly overestimated in denser and/or older stands. Node was included as a random effect to capture unaccounted spatial variation resulting from unobserved explanatory variables.

**FIGURE 4:**
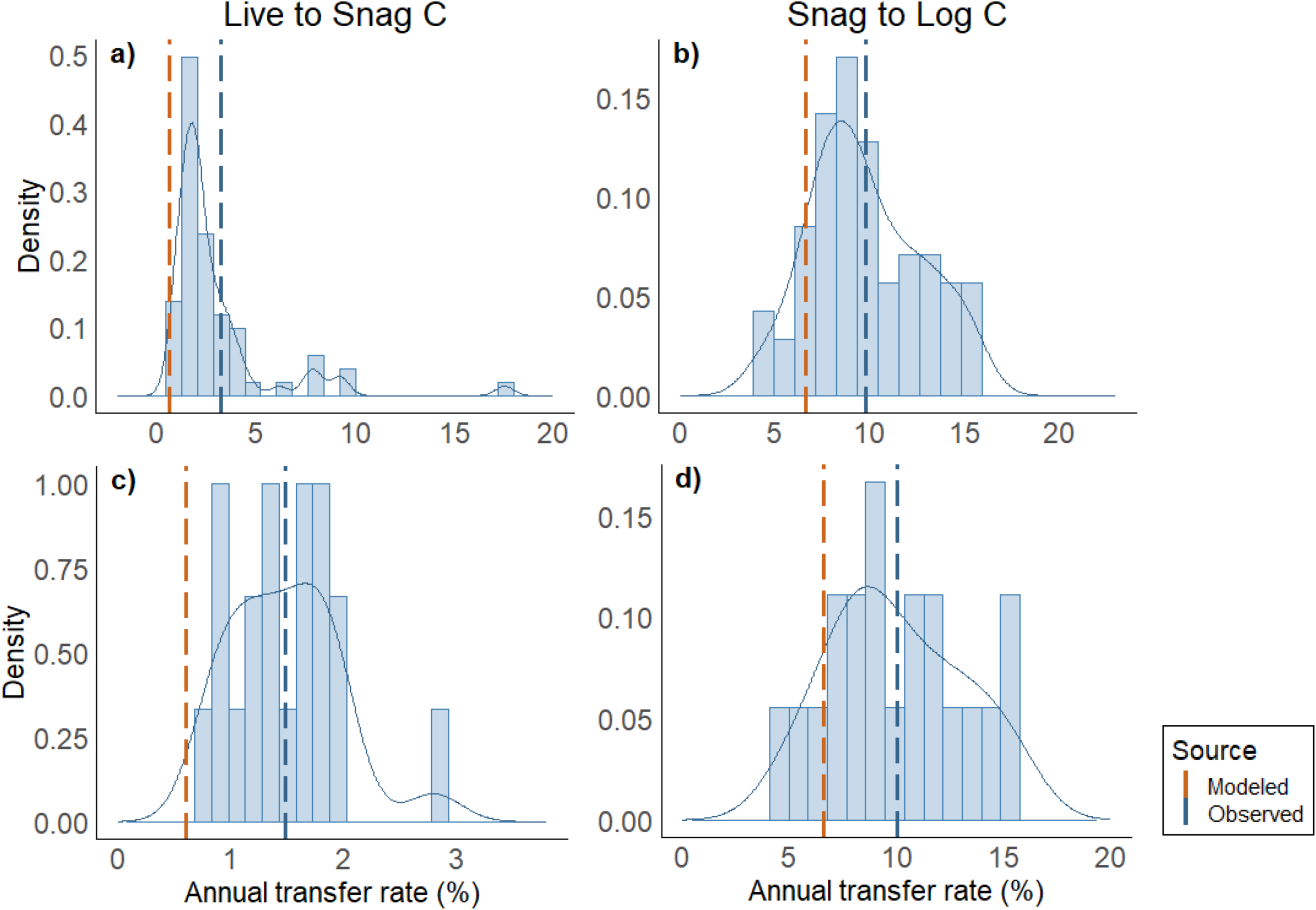
Annual carbon transfer rates between forest carbon pools, based on data from the Climate Impacts on the Productivity and Health of Aspen (CIPHA) project. Panels show: (a) transfer from live biomass to snag pool, and (b) from snag to log pool, averaged across the 64 CIPHA stands included in the present study; and (c) transfer from live biomass to snag pool, and (d) from snag to log pool, averaged across the 17 CIPHA stands in the present study that experienced no secondary disturbances (defined as a minimum 10% reduction in aboveground biomass between survey years). Annual transfer rates were calculated by dividing observed transfers between measurement years by the 4‐year survey interval. Orange dashed lines denote default CBM‐CFS3 C transfer rates, and blue dashed lines denote observed annual averages.

CBM‐CFS's underprediction of the observed stem snag C stocks indicated a potential underestimation of C transfer rates from living biomass to snags and/or overestimation of C transfer rates from snags to logs in the model. Observed C transfer rates from live biomass to snags averaged 3.20% per year (± 0.45% SE), which was substantially higher than the model's default assumption rates of 0.6% per year in the Subhumid Prairies and Taiga Plains, and 0.5% per year in the Boreal Plains (Figure 4a). Similarly, the observed C transfer rates from snags to downed logs averaged 9.77% per year (± 0.36% SE), significantly greater the model's rate of 6.6% per year (Figure 4b).

To further evaluate these rates, we filtered stands to exclude those that had experienced at least a 10% reduction in aboveground biomass between survey years, isolating 17 stands without secondary disturbances. In this subset, observed C transfer from the live to snag pool averaged 1.48% (± 0.11% SE) (Figure 4c) and transfer from snags to downed logs averaged 10.02% (± 0.69% SE) (Figure 4d), both still exceeding the model's default rates. A Mann–Whitney *U* test showed that the average live to snag C transfer rate was significantly higher when including all stands compared to filtered stands (*p* < 0.001). However, no significant difference was found for snag to log C transfer rates between the two groups.

### Forest floor carbon pool

**FIGURE 5:**
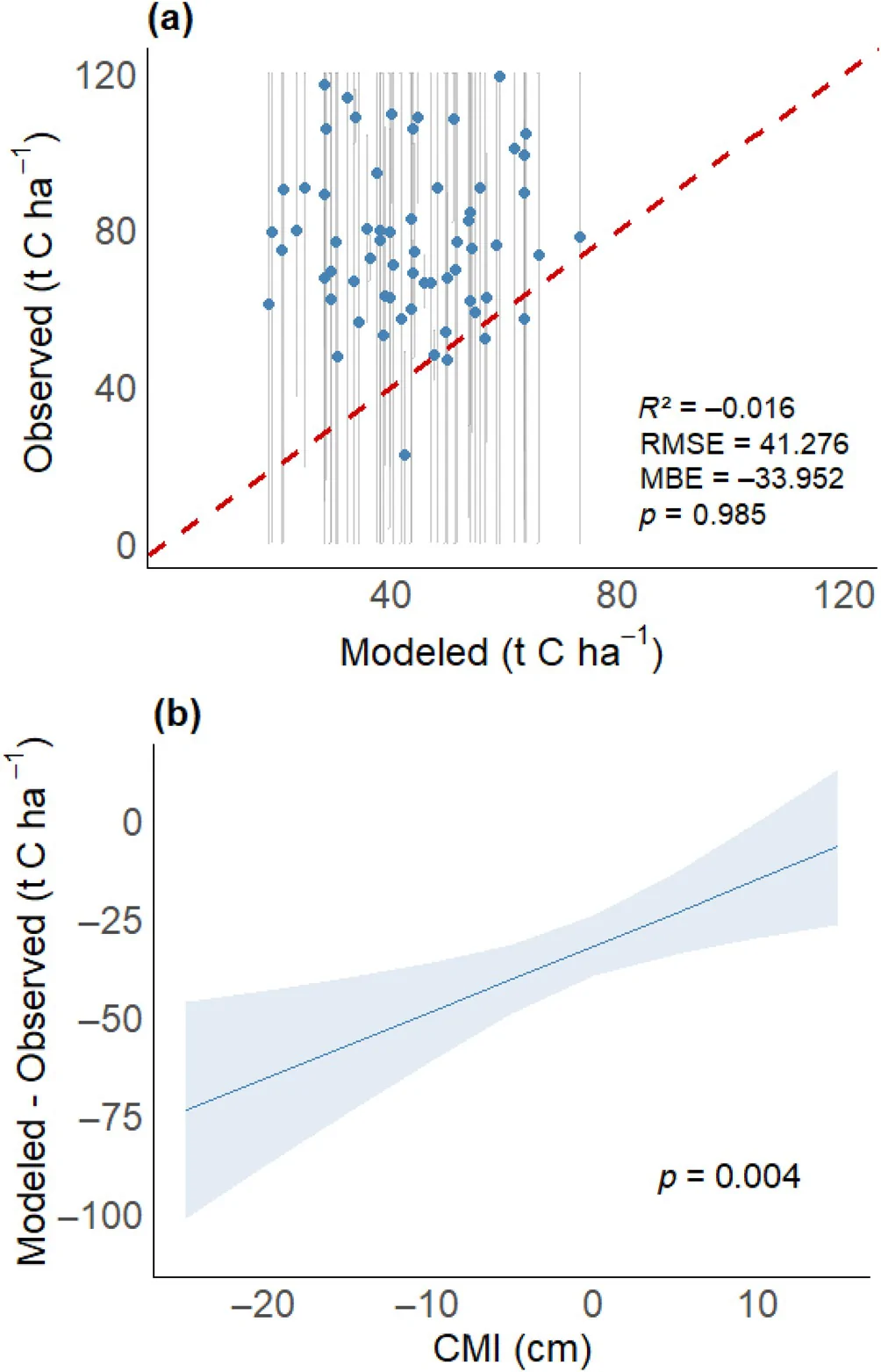
(a) Relationship between CBM‐CFS3 simulated and observed forest floor carbon (C) stocks for 64 stands from the Climate Impacts on the Productivity and Health of Aspen project. Observed soil data were collected in 2002. Each point represents the mean forest floor C stock for a stand, based on two to six samples per stand, and error bars represent 95% CI. The dashed 1:1 line denotes perfect agreement between modeled and observed values. A simple linear regression revealed no significant relationship (*p* = 0.985). Model performance evaluated using a combination of squared correlation coefficient between modeled estimates and observations (*R* ^2^), root mean square error (RMSE), and mean bias error (MBE). (b) Marginal effect of 20‐year averages of climate moisture index (CMI) was a significant predictor of variation in CBM‐CFS3 residuals in the forest floor C pool, derived from a linear mixed‐effects model.

CBM‐CFS3's performance at simulating C stocks in the forest floor was worse compared to that for aboveground biomass and snag pool. There was no significant correlation between CIPHA estimates and simulations; RMSE was 41.28 t C/ha, and CBM‐CFS3 on average underestimated forest floor C stocks by 33.95 t C/ha (Figure 5a). CBM‐CFS3 residuals were best explained by the linear mixed model, with 20‐year averages of CMI as a fixed effect and node as a random effect. CMI explained 21.8% of the variance in the residuals, and together with random effects, the model explained 65.2% of the variance, suggesting that the location‐specific characteristics not captured by the fixed effects played a large role in explaining model residuals. A positive relationship was found between CMI and model residuals (Figure 5b). In stands that experienced drought (negative CMI), the CBM‐CFS3 tended to underpredict forest floor C stocks, with the magnitude of underpredictions increasing with drought severity.

### Mineral soil carbon pool

**FIGURE 6:**
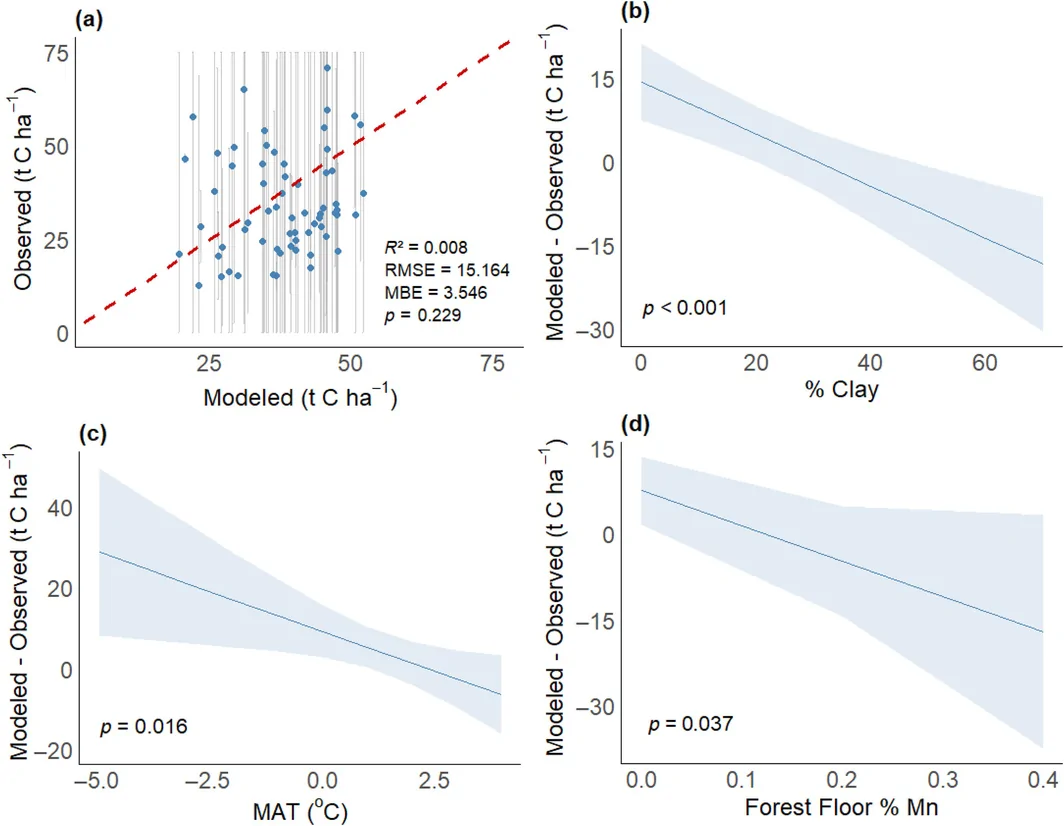
(a) Relationship between CBM‐CFS3 simulated and observed mineral soil organic carbon (SOC) stocks for 64 stands from the Climate Impacts on the Productivity and Health of Aspen project, down to a soil depth of 10 cm. Modeled SOC was scaled to the top 10 cm using estimated depth distribution of C according to Shaw et al. (2018)'s upland forest soil database (see section *Comparison pool* **s** for further details). Observed soil data were collected in 2002. Each point represents the mean mineral SOC stock for a stand, based on two to six samples per stand, and error bars represent 95% CI. The dashed 1:1 line denotes perfect agreement between modeled and observed values. A simple linear regression revealed no significant relationship (*p* = 0.229). Model performance evaluated using a combination squared correlation coefficient between modeled estimates and observations (*R* ^2^), root mean square error (RMSE), and mean bias error (MBE). Marginal effect of (b) soil texture, (c) 20‐year averages of mean annual temperature (MAT), and (d) manganese concentration in the forest floor were significant predictors of variation in CBM‐CFS3 residuals in the mineral SOC pool, derived from a linear mixed‐effects model.

Similar to forest floor C stocks, simulated C stocks for top 10 cm of mineral soil were not significantly correlated with the CIPHA estimates (Figure 6a). The RMSE was 15.16 t C/ha, and C stocks were, on average, overestimated by 3.55 t C/ha. Variation in the CBM‐CFS3 model residuals was best explained by a linear mixed model with mineral soil clay content and forest floor residuals as fixed effects and node as a random effect.

The fixed effects explained 27.2% of the variance in the CBM‐CFS3 residuals, and together with random effects, 78.3% of variance was explained, highlighting the strong influence of site‐specific characteristics on topsoil SOC stocks. CBM‐CFS3 underestimated mineral soil C stocks at clay‐rich sites and overestimated them at the sites with low clay content (Figure 6b). A significant negative relationship was found between 20‐year averages of MAT and the mineral topsoil error. The mineral C stocks were overestimated where MAT was low and underestimated at the warmer sites (Figure 6c). In addition, manganese concentration in the forest floor was significantly negatively related to mineral soil residuals, with CBM‐CFS3 underestimating mineral soil C stocks in stands with higher forest floor %Mn (Figure 6d).

## DISCUSSION

This study evaluated the performance of the CBM‐CFS3 at simulating C stocks in aspen‐dominated forests by comparing the simulated values against measurements collected during the CIPHA project and identified areas for further model improvement. Model validation across a variety of climatic and edaphic conditions is rare due to the limited availability of ground plot data that contain measurements of C stocks in the living and dead organic matter pools. The CIPHA dataset, spanning multiple provinces over 25 years, thus provides a valuable resource for model validation. Our evaluation focused on aboveground biomass, snag, forest floor, and mineral soil carbon pools, identifying both strengths and limitations of the CBM‐CFS3 at simulating C dynamics in these pools. Overall, while some variability in the residuals was attributed to stand density, climatic conditions, soil texture, and chemical composition, most variability remained linked to unmeasured site‐specific characteristics.

### Aboveground biomass

The CBM‐CFS3 performed well at simulating C stocks in the aboveground biomass, explaining over 88% of the variation in the field data. Our findings echo results of previous evaluations of the CBM‐CFS3 against National Forest Inventory ground plots, which also reported strong model performance for this pool (Shaw et al., 2014). Still, stand density and age emerged as significant predictors of model residuals, accounting for 7.4% of their spatiotemporal variation. Specifically, the model tended to increasingly underestimate aboveground biomass C stocks with increasing stand density and age. Within CBM‐CFS3, aboveground biomass is estimated from merchantable volume, provided as model input, using empirical equations specific to jurisdiction, ecozone, and leading tree species (Boudewyn et al., 2007; Kull et al., 2019; Kurz et al., 2009). Merchantable volume was calculated from CIPHA measurements, and the residuals of Equation 1 showed a significant correlation with stand density (Appendix S1: Figure S2c), but not age. This density‐dependent bias in volume was likely the main cause of the density‐dependent bias in aboveground biomass C stocks. The additional effect of stand age on the bias in CBM‐CFS3's aboveground biomass predictions may have resulted from age‐driven variation in biomass allocation patterns that empirical volume‐to‐biomass conversion equations do not capture.

To further evaluate this premise, we compared the proportions of total biomass stored in stemwood calculated from CBM‐CFS3 output to those estimated using tree‐level allometric equations and observed CIPHA tree metrics. Substantial differences were identified between modeled and observed stemwood proportions (Appendix S1: Figure S3a), which were linearly related to stand age (Appendix S1: Figure S3b). Interestingly, CBM‐CFS3 tended to increasingly overestimate the stemwood proportion with stand age—a pattern that is opposite to the expected one, as CBM‐CFS3 tended to increasingly underestimate the aboveground biomass with stand age (Figure 2c). This apparent contradiction may arise from the indirect influence of stand density on aboveground biomass residuals, as stand density declined exponentially with stand age (Appendix S1: Figure S3c). Consequently, the observed relationships between residuals, stand age, and stemwood allocation likely reflect underlying nonlinear interactions among these predictors.

Age‐related processes, such as self‐thinning, can alter stand density and tree growth, modifying biomass allocation patterns in ways that are currently not represented in the model's allometric equations. In low‐density stands, individual trees face less competition for resources such as light, water, nutrients, and space, which often allows for larger and broader crowns (Long & Smith, 1984), and for a greater allocation of biomass to branches and foliage relative to stems (Truax et al., 2018). As CBM‐CFS3 applies age‐independent volume‐to‐biomass allometric relationships, it can overestimate stemwood, and therefore aboveground biomass carbon, in older low‐density stands where a given stand volume may consist of fewer, larger trees with different biomass allocation patterns. Conversely, in young, dense stands, intense competition for light and resources typically promotes greater investment in vertical stem growth at the expense of crown and branch development, producing narrower crowns and higher stem‐to‐branch ratios (Li et al., 2022; Truax et al., 2018). This pattern may have contributed to CBM‐CFS's underestimation of stemwood proportions, and hence aboveground biomass C stocks, in these younger denser stands. Our results suggest that incorporating age into empirical volume‐biomass relationships as well as stand density into merchantable volume estimation could further improve CBM‐CFS's performance at simulating aboveground biomass C stocks in aspen‐dominated stands.

### Snags

Standing dead trees and their persistence are a major component of forest C stocks, particularly in the years following disturbance (Hilger et al., 2012; Stinson et al., 2011; Woodall et al., 2008). Our evaluation showed that the CBM‐CFS3 explained 43% of the variation in snag C stocks and, on average, slightly underestimated their magnitude. The model represents snag C dynamics as a balance between C inputs from region‐specific background and disturbance‐driven tree mortality and losses via snag decay, which is dependent on temperature and fall (Kurz et al., 2009; Kurz & Apps, 1999). Therefore, the average underestimation of the snag C stocks could have been due to underestimated mortality, overestimated fall rates (we ignore the effect of snag C decay as fall rates are nearly double those of decay (Hararuk et al., 2017)), or both.

At CIPHA stands, CBM‐CFS3 consistently underestimated both C inputs to and C losses from the snag pool when compared to observed data (Figure 4). As expected, filtering the observed data to include only stands without secondary disturbance resulted in significantly lower average mortality, yet this value was still more than twofold greater than the model's default value. Similarly, reported annual mortality rates in aspen forests, ranging from 0.08% to 8.2%, were substantially higher than the CBM‐CFS3's default rates of 0.5%–0.6% for the ecozones in this study (Lee, 1998). In our CBM‐CFS3 simulation, we applied a calibrated snag annual fall rate of 6.6% (Hararuk et al., 2017), approximately double the model's default value. Even so, the observed rates of snag fall in these aspen‐dominated stands were higher than the value calibrated against the National Forest Inventory ground plot observations across all species. Snag fall rate was not substantially affected by disturbances at CIPHA plots, as indicated by no significant change in the average values when disturbed plots were excluded from the analysis. Previously reported snag fall rates vary widely, ranging from 7.04% per year (Hilger et al., 2012) to 9%–21% per year (Hogg & Michaelian, 2015; Lee, 1998) and exceed the CBM‐CFS's default value of 3.2% per year.

Snag persistence and carbon retention depend on various tree‐ and stand‐level factors (Aakala et al., 2024). Greater stand density and rooting depth, for example, may enhance snag persistence by buffering wind exposure (Peltola, 2006). Wood decay rates, influenced by factors such as temperature, soil moisture, decomposer activities, tree allometry, and wood quality, can also dictate snag fall rate (Oberle et al., 2018; Zanne et al., 2015). With many drivers of snag fall dynamics, Shaw et al. (2014) reported high uncertainties in the standing deadwood pools generated by CBM‐CFS3, often with overestimations, though no significant model bias was found in trembling aspen stands.

In our analysis, CBM‐CFS3 bias in the aboveground biomass C stocks explained 2.8% of model residual variance, with the explained variance increasing to nearly 21% when local site conditions were included (Appendix S1: Table S3). CBM‐CFS3 underpredicted snag C stocks in stands where aboveground biomass C was overestimated, which were typically low‐density stands, and overpredicted snag C stocks in stands where aboveground biomass C was underestimated, which generally corresponded to high‐density stands. The underlying causes for over‐ versus underprediction likely differed. At low‐density stands (up to 500 stems/ha), there was as much or more C stored in snags as in aboveground biomass at CIPHA plots (Appendix S1: Figure S4), indicating increased mortality from a recent disturbance. Several low‐density stands showed significant increases in snag C stocks in years not directly associated with recorded disturbance events, many of which occurred in 2008. Increased mortality around 2008 was likely a lagged effect from the severe 2001–2002 drought in the region (Bonsai & Wheaton, 2005; Hogg et al., 2008) and subsequent aspen decline. Such a response aligns with delayed sudden aspen decline, in which multi‐year lag effects of moisture stress and other underlying factors can peak even 6 years later (Worrall et al., 2010). Thus, CBM‐CFS's underestimation of snag C in the low‐density stands, where aboveground biomass C was also overestimated, was likely due to its lack of representation of drought‐induced lagged tree mortality. The overestimation of snag C stocks at the high‐density stands was likely due to the more than two‐fold lower fall rates in the CBM‐CFS3 noted earlier.

Past studies have recommended several refinements to snag pool modeling, including using higher, species‐specific, and dbh‐specific fall rates, applying stem biomass‐based rather than stem counts‐based carbon transfer rates, and accounting for stand age effects (Hagemann et al., 2010; Hilger et al., 2012; Lee, 1998), although our analysis did not detect significant age‐related influences on CBM‐CFS3 biases in snag C stocks. Building upon these recommendations, we also highlight the value of incorporating lagged mortality from drought events and increasing snag fall rates to further improve the CBM‐CFS3's ability to represent this pool. The observed fall rates were much higher than both the default and calibrated values, and we note that methodological limitations may have even slightly underemphasized these estimates, especially in stands with severe disturbances where snag phases may be brief or absent. We calculated snag fall as the proportion of snag biomass C lost by the next sampling interval, 4 years later. Such long intervals can miss instances where live trees become snags and then logs between surveys, resulting in an overestimation of snag persistence.

### Forest floor

We found that CBM‐CFS3 did not perform well at capturing magnitudes and spatial variability of the organic layer C stocks in the aspen‐dominated stands. Although our model runs were generated using the parameters informed by the NFI ground plot observations (Hararuk et al., 2017), we demonstrate that substantial model modifications are still needed to produce reliable carbon estimates in this pool. Despite small forest floor sample sizes at CIPHA plots, with two to six samples per stand, and high variability around observed means, the model consistently underestimated forest floor C stocks. This contrasts with Shaw et al. (2014), who found that CBM‐CFS3 overestimated forest floor C stocks in trembling aspen stands, attributing this to faster litter (Alban & Pastor, 1993) and dead wood (Angers et al., 2012) decay rates compared to other common tree species. This difference likely reflects changes in parameter values: while Shaw et al. (2014) used the default CBM‐CFS3 parameter set, we used a parameter set calibrated against NFI ground plots observations. The systematic underestimation of the forest floor C could have resulted from the short default fire return intervals in the model, which ranged from 75 to 125 years at CIPHA plots, as fires burn a fraction of forest floor. To explore this, we set the fire return intervals to values more representative of aspen stands in each province (301.9 to 387.4 years; Nlungu‐Kweta et al., 2017). However, this adjustment resulted in only a modest reduction in the average underprediction (36.70 vs. 33.96 t C/ha).

Analysis of the model residuals revealed the importance of site moisture conditions: 20‐year averages of CMI explained 21.8% of residual variability, with drier sites showing larger underestimations of forest floor C stocks. Droughts can affect both C input rates into the forest floor and its decay rates. Drought stress may trigger earlier and increased litterfall as trees shed leaves to reduce water loss through transpiration (Frisko et al., 2024; Limousin et al., 2009), increasing organic inputs to the forest floor. In addition, drought events have been reported to cause widespread aspen dieback (Hogg et al., 2008; Michaelian et al., 2011), which in turn increases branch litterfall from the dead trees. Moisture is a key control on organic matter decomposition, with decay rates increasing under moist conditions and decreasing under dry conditions (Prescott, 2010; Walse et al., 1998; Wickland & Neff, 2008). Limited soil water availability reduces nutrient diffusion and increases soil water tension, which may prompt microbial decomposers to adopt drought response strategies such as decreased activity or even dormancy (Manzoni et al., 2014; Sierra et al., 2017), slowing decomposition and prompting organic matter accumulation. The effects of drought on C input to the forest floor are not yet represented in CBM‐CFS3. However, ongoing efforts to develop climate‐sensitive growth and yield models may allow incorporation of drought impacts into merchantable yield estimates (Metsaranta et al., 2024), which would cascade into litterfall estimates. Previous analyses from the Canadian Intersite Decomposition Experiment (CIDET) highlight the need to account for moisture effects on litter decay within CBM‐CFS3 (Smyth et al., 2011), and we reinforce this recommendation as essential for improving representation of organic layer C dynamics.

Still, unobserved site‐specific conditions accounted for an additional 43% of residual variation after correcting for CMI effects. A large‐scale analysis of the controls on forest floor C stocks in the boreal forests revealed depth to water table as the most important predictor (Hagenbo et al., 2025), which could affect both C input and decomposition. Other factors that may affect forest floor C stocks include disturbance history and presence of earthworms. Past fires that were not accounted for in the CBM‐CFS3 may contribute to forest floor C stocks overestimation by removing forest floor C that the model does not simulate, while past insect outbreaks could contribute to underestimation by creating unrepresented C input pulses. Earthworms, native or exotic, mix and aerate the forest floor and accelerate decay (Alban & Berry, 1994; Buchkowski et al., 2024; Lubbers et al., 2013), thus potentially contributing to overestimations at some sites. Detailed site history, along with additional measurements such as earthworm biomass, would help identify sources of residual variability in the model. Because forest floor data at CIPHA plots were collected more than 20 years ago and the sites have been continuously monitored since, remeasuring organic layer C stocks would provide an opportunity to evaluate the model's ability to simulate C dynamics in this pool once the detailed site history is accounted for. For example, the model could be initialized with the first set of measurements, run forward in time, and then assessed against the second set of measurements to determine the amount of variability in the observations that could be attributed to site history.

### Mineral soil

Similar to the forest floor, CBM‐CFS3 did not capture spatial variability in mineral soil carbon estimates. However, the model bias was much smaller (3.55 t C/ha) and, unlike the forest floor results, the CBM‐CFS3 tended to slightly overestimate C stocks. Model evaluation was further complicated by high variability in observed data, partly due to small sample sizes per stand (Table 1). Around 27% of residual variation was explained by clay content, MAT, and organic layer Mn concentration (listed in order of importance; Appendix S1: Table S5), and about 50% by unobserved site‐specific conditions.

Clay content was the most important fixed effect, with the CBM‐CFS3 overestimating mineral C stocks in low‐clay soils and underestimating in high‐clay soils (Figure 6b). Clay and fine silt particles (<20 μm) promote aggregate formation and organo‐mineral interactions, physically protecting soil organic matter and enhancing C storage potential (Carter et al., 2003; Hassink, 1997; Lützow et al., 2006; Singh et al., 2018). They also increase the threshold for C accumulation, as described by the carbon saturation concept (Stewart et al., 2007). Furthermore, fine‐textured soils typically sustain higher microbial biomass and activity through higher cation exchange capacity and greater water and nutrient availability (Callesen et al., 2003; Wei et al., 2014). Their greater microbial community diversity and microbial substrate use (Domeignoz‐Horta et al., 2020; Seaton et al., 2020; Wei et al., 2014) often lead to less carbon lost through respiration and more retained in soils (Angst et al., 2021). Despite the complexity of interacting processes and confounding factors not assessed here, the overarching pattern remains that finer soils retain more carbon, and adopting soil texture as one of the controls of soil C decay will improve simulation of mineral soil C dynamics.

Mean annual temperature was the second most important predictor among fixed effects, even though temperature effects on decay are represented in the CBM‐CFS3 (Hararuk et al., 2017; Kurz et al., 2009), where decay rates accelerate under warmer conditions. Model evaluation showed that mineral soil C stocks were overestimated at cold sites and slightly underestimated at warm sites (Figure 6c), suggesting that the temperature sensitivity of decomposition, or Q10, was too high for aspen‐dominated sites. Q10 values tend to decrease with increasing substrate quality (Conant et al., 2008; Hartley & Ineson, 2008; Wetterstedt et al., 2010), whereas CBM‐CFS3 currently assumes a single value for each pool, and we used the values calibrated across forest stands in Canada (Hararuk et al., 2017), 67.8% of which were coniferous (Natural Resources Canada, 2025). Because aspen‐dominated stands have lower soil C:N ratios, which could indicate higher substrate quality compared to white and black spruce‐dominated stands (Hannam et al., 2004; Légaré et al., 2005), the Q10 values used in this study were likely too high.

Mn concentration was the least important, but significant predictor of CBM‐CFS3 mineral soil C residuals, with higher Mn concentrations associated with greater underpredictions of mineral soil C stocks. Mn is often a limiting nutrient for microbial activity in forest soils and plays a key role in organic matter decomposition. Previous studies generally report a negative relationship between manganese availability and C stocks in the forest floor (Hagenbo et al., 2025), attributed to manganese‐dependent fungi accelerating litter oxidation and humus turnover in boreal forests (Stendahl et al., 2017). While mineral soil Mn concentration was not measured in this study, sites with higher forest floor Mn may also have higher Mn concentration in the mineral soil due to processes like bioturbation or leaching. However, our analysis indicated the opposite trend, suggesting that other factors, such as soil depth, soil properties, site characteristics, and the fungal to bacteria biomass ratio, may buffer or mask manganese's typical effects. For example, Possinger et al. (2022) found that manganese's role shifts with soil depth, promoting oxidation near the surface through fungal activity but reducing decomposition in deeper layers where bacteria dominate and SOC is more mineral associated. Alternatively, higher Mn concentration could have increased microbial activity and forest floor C decay, increasing the input of microbial compounds that form stable organic matter (Bradford et al., 2013; Cotrufo et al., 2013) into the mineral soil, thus increasing mineral C stocks more than predicted by CBM‐CFS3. Further research is needed to clarify Mn's role in forest floor and mineral soil C dynamics at these stands to refine model modifications.

Similar to the forest floor, most of the variability in the CBM‐CFS3 mineral soil C residuals (about 50%, Appendix S1: Table S5) was attributed to unobserved site‐specific conditions. While earthworms also influence mineral soil C stocks, their effect contrasts with that on the forest floor: they tend to contribute to higher mineral C stocks via mixing organic layer C into the mineral layer and facilitating C stabilization in earthworm casts and organo‐mineral complexes (Addison, 2009; Ferlian et al., 2020). Other unobserved conditions that could potentially explain variation in the mineral C stocks in the boreal region are variables associated with soil moisture regime, such as drainage class, parental material, and slope (Dalsgaard et al., 2016; Hagenbo et al., 2025). Lastly, the large effect of site‐specific conditions on the model residuals may also reflect variation in vertical C storage patterns within the mineral profile. In this study, to make model estimates of mineral C stocks comparable to the observed ones, we used an empirical equation (Equation 2) to determine the fraction of the total mineral C stock stored in the top 10 cm. However, the uncertainty around this estimate was high (see Appendix S1: Fig. S1). Increasing the sampling depth and explicitly characterizing soil profile would improve our understanding of the roles of soil drainage and earthworms on mineral C stocks as well as help determine whether the drivers of topsoil C stocks identified here also affect C dynamics in deeper layers.

### Limitations

The CIPHA data central to this study were collected in the context of increasing drought‐related stress and climate‐induced disturbances on aspen forests. These changes are reflected in year‐to‐year shifts in aboveground productivity and mortality, which ultimately influence C dynamics in downstream forest floor and mineral soil pools. One key limitation of this dataset for the purpose of carbon monitoring is that soil samples from both the forest floor and mineral layers were collected during only one survey year, around the time of study establishment. As all stands were initially healthy at the time of study establishment, it would be valuable to resample soils in these plots, especially those that have experienced significant change in the aboveground biomass, to observe how belowground C stocks have evolved in response to that change. Repeated soil sampling would improve temporal resolution and refine model predictions regarding the timing and rate of carbon decay and its transfer between pools. Results in the forest floor and mineral soil pools are also limited by the small sample size, contributing to high variance in mean C stock estimates. Increasing the number of samples in future work would enhance statistical confidence and improve our ability to quantify the effects of key variables.

Additionally, future work may benefit from incorporating downed wood sampling in field surveys. We could track tree transitions from live to snag and snag to downed wood from survey to survey with the current data, but the lack of direct measurements of downed wood limited our ability to assess decay rates and downstream carbon transfers. As a result, it is unclear whether model biases in the dead organic matter pool primarily stem from decay and transfer from live‐to‐snag, snag‐to‐log, or log‐to‐forest floor. While tracing tree transitions provided a general representation of carbon flow rates, incorporating direct measurements of dead wood, including decay class for snags and logs, could clarify the model's performance in simulating dead organic matter pool dynamics. Downed wood serves as the intermediate carbon pool between snags and the forest floor, therefore better representation of this pool could clarify and reconcile upstream factors lending to CBM's current underperformance in simulating forest floor and soil carbon stocks. Including these measurements in future datasets would help fill this gap and refine dead organic matter dynamics within the model. Field data also carried varying degrees of uncertainty due to coordination across multiple provinces and field crews. While detailed sampling protocols and trainings were in place, variation in data collection conducted by different field crews may have introduced some mensuration errors. That said, as the CIPHA study is an ongoing monitoring project, current limitations can be addressed.

Analysis of the model residuals showed that most of their variabilities were attributed to unobserved site‐specific conditions, particularly in the forest floor and mineral soil. Variables most likely to explain additional residual variation are earthworm presence and biomass, along with parameters associated with soil moisture regime, such as drainage class, parental material, and slope. Measuring these variables in the future may provide further insights into controls of the dead organic matter dynamics in the aspen‐dominated stands. For mineral soils, increasing sampling depth and determining the depth of the soil profile would remove the uncertainty introduced by Equation (2) and facilitate evaluation of the effects of earthworms and moisture regime on C dynamics in this pool.

A broader limitation of the CBM‐CFS3 lies in its reliance on empirical growth and yield models that lack climate sensitivity, limiting its ability to simulate forest growth and C dynamics under changing climate conditions and extreme climatic events (Metsaranta et al., 2024). While this aspect was not addressed here, future work should explore ways to integrate climate sensitivity into aboveground biomass dynamics to ensure that improvements identified in the current and future studies can be meaningfully incorporated under variable environmental conditions.

Our work aimed to build upon existing strengths of the model by evaluating predicted C stock in various pools against detailed ground plot data. This study identified clear opportunities to further improve model accuracy, particularly by refining dead organic matter transfer rates, incorporating site‐specific variables like soil texture and moisture, and validating predictions against more frequent and spatially resolved field data. This study contributes to the continued refinement of the CBM‐CFS3 for increasingly accurate and realistic projections, ultimately enhancing its value as a tool for national and international carbon accounting objectives.

## AUTHOR CONTRIBUTIONS

Oleksandra Hararuk conceived and designed the model evaluation study. Michael Michaelian led the data collection and curated the CIPHA dataset. Derek Sattler developed merchantable yield curves from CIPHA measurements. Nicole Lau ran the CBM‐CFS3, analyzed model performance with guidance from Oleksandra Hararuk, and led the drafting of the manuscript. All authors contributed to editing and revisions of the manuscript.

## CONFLICT OF INTEREST STATEMENT

The authors declare no conflicts of interest.

## Supporting information


Appendix S1:

## Data Availability

Data (Lau, 2025) are available in Zenodo at https://doi.org/10.5281/zenodo.20188296.
