# Supplementary material for: Improving carbon modeling in aspen‐dominated forests: Lessons from CBM‐CFS3 evaluation against long‐term field data
Source: Ecol Appl. 2026 Sep 18;36(6):e70307. doi: 10.1002/eap.70307 (PMC13586845; doi:10.1002/eap.70307)
Supplement: Supplementary file 1 [file EAP-36-e70307-s001.pdf]

Appendix S1

Improving carbon modeling in aspen-dominated forests: Lessons from CBM-CFS3 evaluation against long-term field data

Nicole Lau, Derek Sattler, Michael Michaelian, Oleksandra Hararuk

Ecological Applications

Table S1. Estimates for parameters in Eq. 1 for stands dominated by trembling aspen and located in Boreal Plains, Taiga Plains, or Subhumid Prairies.

	Trembling aspen			
Parameter	*Estimates*	*SE*	*z-value*	*p-value*
a	-1.008	0.037	-27.21	**<.001**
b	0.0427	0.0007	-59.30	**<.001**
φ	2.71	0.08	33.83	**<.001**
n	2367			
Log-likelihood	4952			
Pseudo-R^2	0.72			

Note. φ characterises the variance of the response: $Var(y_i) = \frac{\mu_i(1-\mu_i)}{1+\varphi_i}$, where μ_i is the *i*th expected value generated by the beta regression. Expected value for proportion of the mineral SOC stored in the top 10 cm is 0.3586 (SD=0.249).

Table S2. Summary table of the linear mixed effects model for CBM-CFS3 residuals in the aboveground biomass carbon pool.

	Aboveground biomass C residuals				
Predictors	*Estimates*	*CI*	*Std. Estimates*	*Std. CI*	*p-value*
(Intercept)	9.74	2.34 – 17.13			**0.010**
Stand density	-0.01	-0.01 – -0.00	-0.33	-0.43 – -0.23	**<0.001**
Stand age	-0.11	-0.18 – -0.04	-0.17	-0.29 – -0.06	**0.004**
**Random Effects**					
σ^2	49.83				
$\tau_{00\ node}$	34.93				
ICC	0.41				
N_{node}	23				
Observations	448				
Marginal R^2 / Conditional R^2	0.074 / 0.456				

Note. Model residuals represented as carbon stocks simulated by the Carbon Budget Model of the Canadian Forest Sector (CBM-CFS3) minus observed. Data from 64 stands as part of the Climate Impacts on the Productivity and Health of Aspen project. Standardized estimate (Std. Estimates) and confidence interval (Std. CI) determined using the get_model_data() function for forest-plot of standardized coefficients in the sjPlot package (v2.8.16; Lüdecke, 2025).

Table S3. Summary table of the linear mixed effects model for CBM-CFS3 residuals in the snag carbon pool.

	Snag C residuals				
Predictors	*Estimates*	*CI*	*Std. Estimates*	*Std. CI*	*p-value*
(Intercept)	-1.06	-2.36 – 0.24			0.111
Aboveground biomass residuals	-0.11	-0.18 – -0.04	-0.17	-0.28 – -0.07	**0.002**
**Random Effects**					
σ^2	33.65				
$\tau_{00\ node}$	7.57				
ICC	0.18				
N node	23				
Observations	446				
Marginal R^2 / Conditional R^2	0.028 / 0.207				

Note. Model residuals represented as carbon stocks simulated by the Carbon Budget Model of the Canadian Forest Sector (CBM-CFS3) minus observed. Data from 64 stands as part of the Climate Impacts on the Productivity and Health of Aspen project. Standardized estimate (Std. Estimates) and confidence interval (Std. CI) determined using the get_model_data() function for forest-plot of standardized coefficients in the sjPlot package (v2.8.16; Lüdecke, 2025).

Table S4. Summary table of the linear mixed effects model for CBM-CFS3 residuals in the forest floor carbon pool.

	**Forest Floor C residuals**				
Predictors	*Estimates*	*CI*	*Std. Estimates*	*Std. CI*	*p-value*
(Intercept)	-31.92	-39.70 – -24.12			**<.001**
CMI (20-year average)	1.68	0.56 – 2.79	0.48	0.16 – 0.80	**.004**
**Random Effects**					
σ^2	207.04				
$\tau_{00\ node}$	257.83				
ICC	0.55				
N_{node}	23				
Observations	64				
Marginal R^2 / Conditional R^2	0.219 / 0.652				

Note. Model residuals represented as carbon stocks simulated by the Carbon Budget Model of the Canadian Forest Sector (CBM-CFS3) minus observed. Observed soil data were collected in 2002 (n = 2–6 per stand). Climate moisture index (CMI) was calculated as average CMI over the 20 years preceding sampling to buffer the influence of potential climate anomalies during the sample year (1982–2002). Data from 64 stands as part of the Climate Impacts on the Productivity and Health of Aspen project. Standardized estimate (Std. Estimates) and confidence interval (Std. CI) determined using the get_model_data() function for forest-plot of standardized coefficients in the sjPlot package (v2.8.16; Lüdecke, 2025).

Table S5. Summary table of the linear mixed effects model for CBM-CFS3 residuals in the mineral soil organic carbon (SOC) pool.

	**Mineral SOC residuals**				
Predictors	*Estimates*	*CI*	*Std. Estimates*	*Std. CI*	*p-value*
(Intercept)	22.97	13.72 – 32.21			**<.001**
% Clay	-0.47	-0.70 – -0.24	-0.44	-0.66 – -0.23	**<.001**
MAT (20-year average)	-3.89	-7.04 – -0.74	-0.41	-0.74 – -0.08	**.016**
Forest Floor % Mn	-61.71	-119.57 – -3.84	-0.19	-0.37 – -0.01	**.037**
**Random Effects**					
σ^2	49.87				
$\tau_{00\ node}$	117.65				
ICC	0.70				
N_{node}	23				
Observations	64				
Marginal R^2 / Conditional R^2	0.272 / 0.783				

Note. Model residuals represented as SOC stocks simulated by the Carbon Budget Model of the Canadian Forest Sector (CBM-CFS3) minus observed, down to a soil depth of 10 cm. Modeled SOC was scaled to the top 10 cm using estimated depth distribution of C according to Shaw et al. (2018)'s upland forest soil database. Observed soil data were collected in 2002 (n = 2–6 per stand). Mean annual temperature (MAT) was calculated as average MAT over the 20 years preceding sampling to buffer the influence of potential climate anomalies during the sample year

(1982–2002). Data from 64 stands as part of the Climate Impacts on the Productivity and Health of Aspen project. Standardized estimates (Std. Estimates) and confidence intervals (Std. CI) determined using the get_model_data() function for forest-plot of standardized coefficients in the sjPlot package (v2.8.16; Lüdecke, 2025).

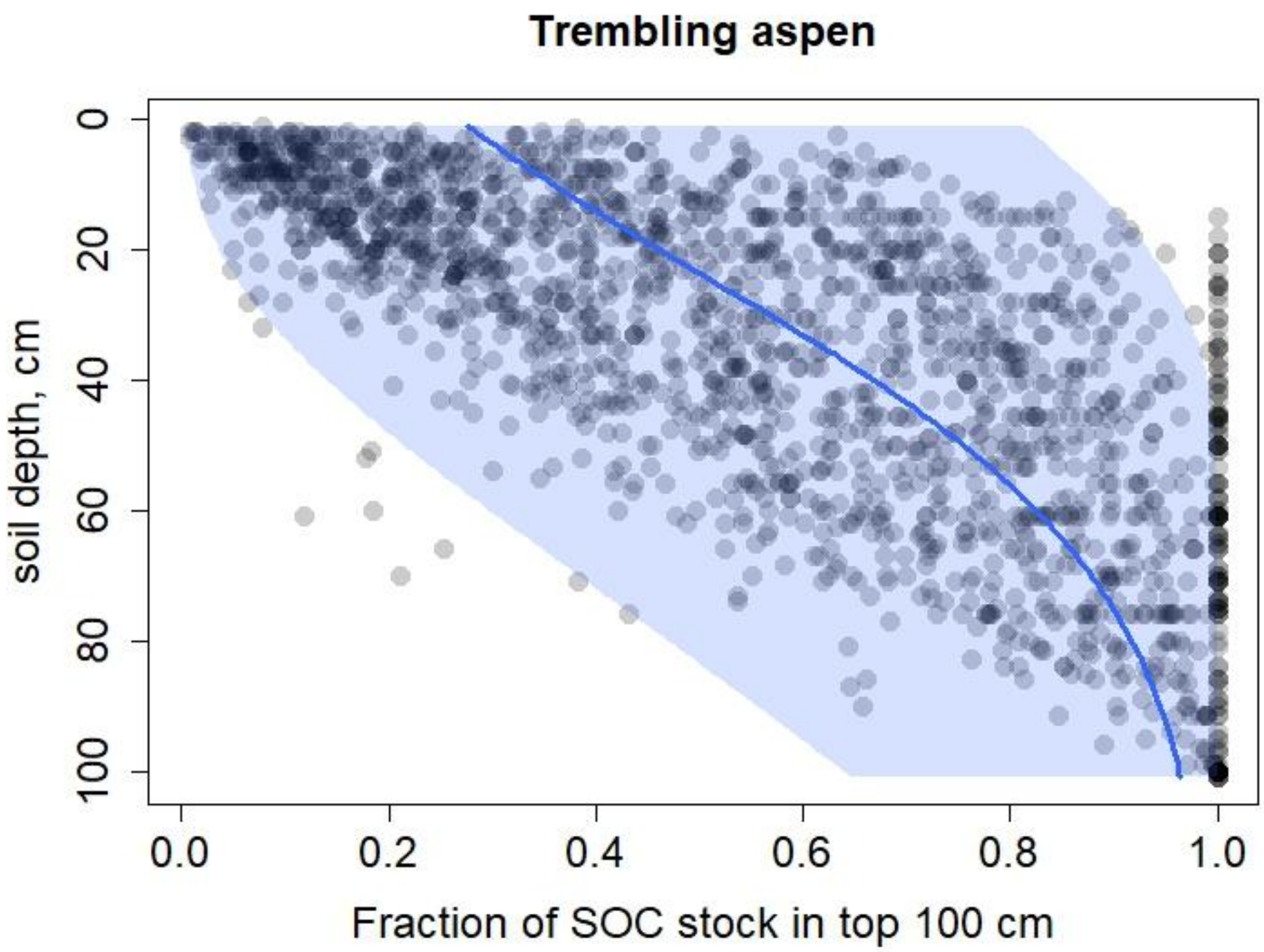

Figure S1. Fit of Eq. 1 to the data from the Canadian upland forest soil profile and carbon stocks database for the stands dominated by trembling aspen (Shaw et al., 2018). The blue line is the expected value, and the shaded area depicts the 95% confidence interval of the prediction.

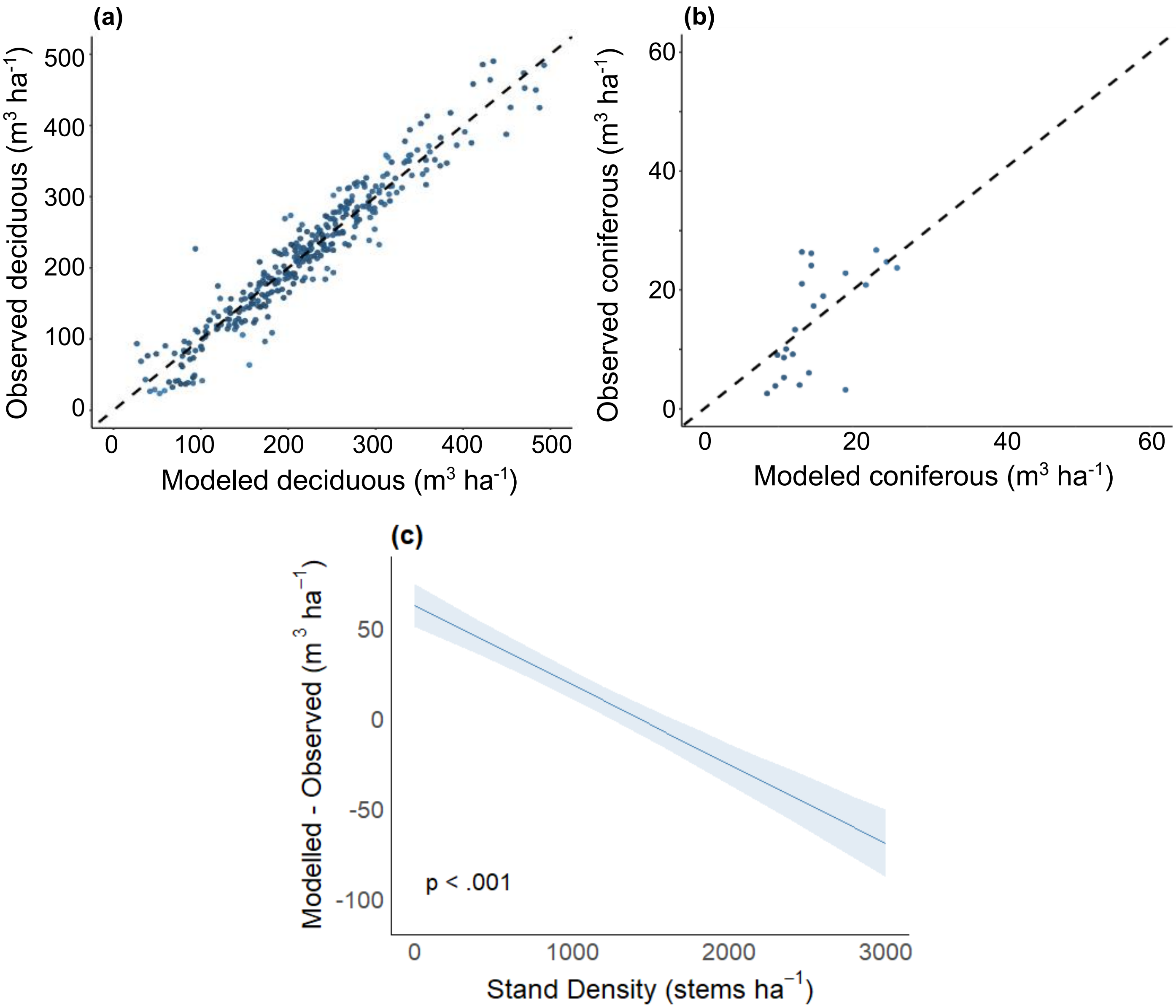

Figure S2. Relationship between modeled and observed volumes of the **(a)** deciduous and **(b)** coniferous components of each stand. Separate yield curves were generated for each component using non-linear mixed effect volume-age models, based on a modified base equation from S. Huang et al. (2009). For the deciduous component only, an anchor point was added to the model fitting dataset at year 200 to prevent unrealistic overprediction of aspen growth. Each point represents a different stand age for 64 stands from the Climate Impacts on the Productivity and

Health of Aspen project. The dashed 1:1 line denotes perfect agreement between modeled and observed values. **(c)** Marginal effect of stand density was a significant predictor of variation in residuals of total live tree volume, accounting for 21.5% of the variance in a linear mixed-effects model. Including node location as a random effect increased the explained variance to 31.3%.

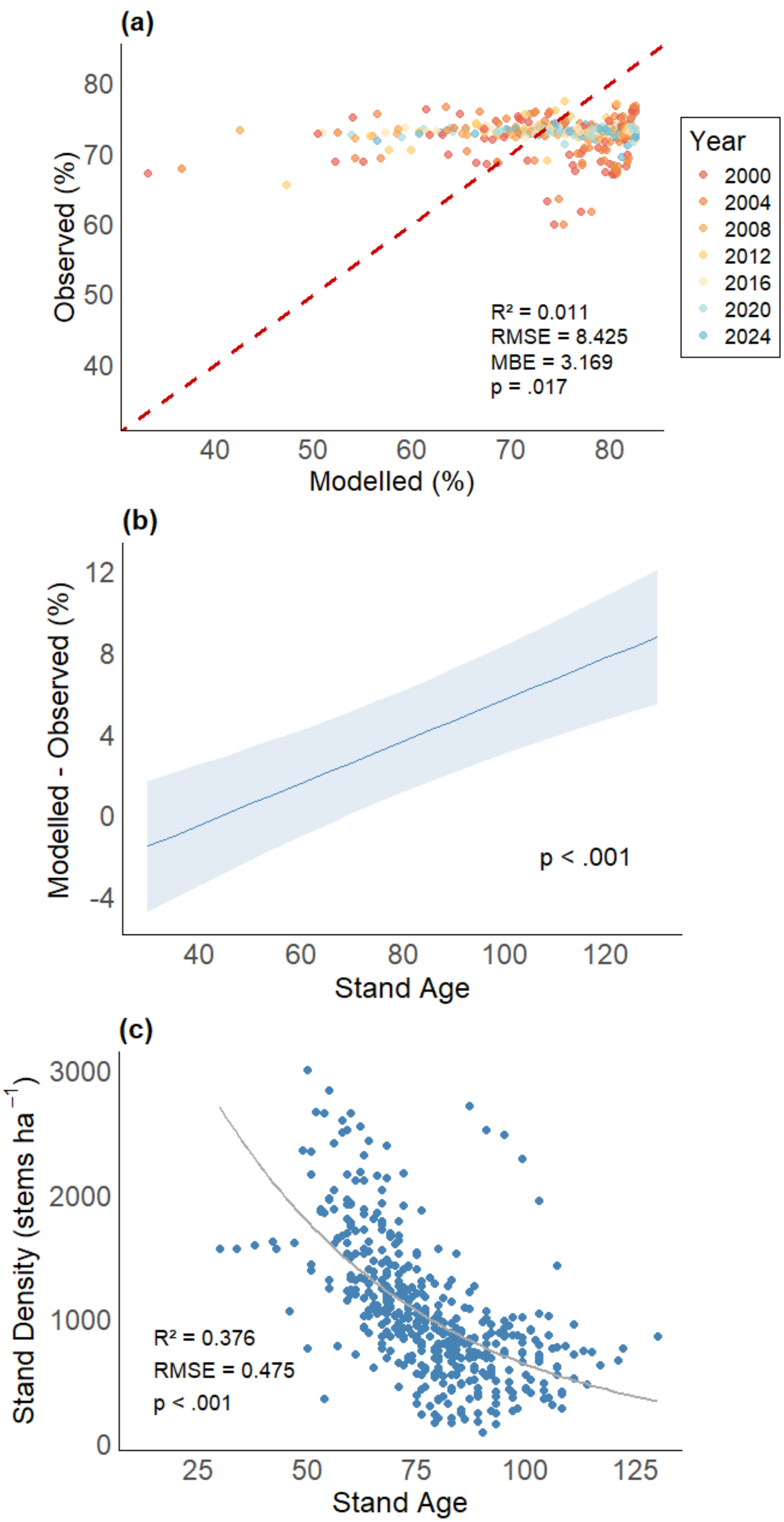
(a)
Observed (%)
Modelled (%)
80
70
60
50
40
40
50
60
70
80
Year
2000
2004
2008
2012
2016
2020
2024
R² = 0.011
RMSE = 8.425
MBE = 3.169
p = .017
(b)
Modelled - Observed (%)
12
8
4
0
-4
40
60
80
100
120
Stand Age
p < .001
(c)
Stand Density (stems ha⁻¹)
3000
2000
1000
0
25
50
75
100
125
Stand Age
R² = 0.376
RMSE = 0.475
p < .001

Figure S3. (a) Relationship between CBM-CFS3 simulated and observed proportion of carbon stored in the stemwood component relative to whole tree carbon for 64 stands sampled in the Climate Impacts on the Productivity and Health of Aspen project. Observed tree biomass components were calculated according to Ung et al. (2008). Points are coloured by the year of field survey. The dashed 1:1 line denotes perfect agreement between modeled and observed values. A simple linear regression revealed a positive but non-significant correlation ($p = .017$). Model performance evaluated using a combination squared correlation coefficient between modeled estimates and observations (R^2), root mean square error (RMSE), and mean bias error (MBE). **(b)** Significant marginal effect of stand age on CBM-CFS3 residuals of stemwood carbon proportion, estimated using a linear mixed effects model. **(c)** A significant negative relationship was found between stand age and stand density ($p < .001$). The solid line represents the fitted general linear model with a log link function.

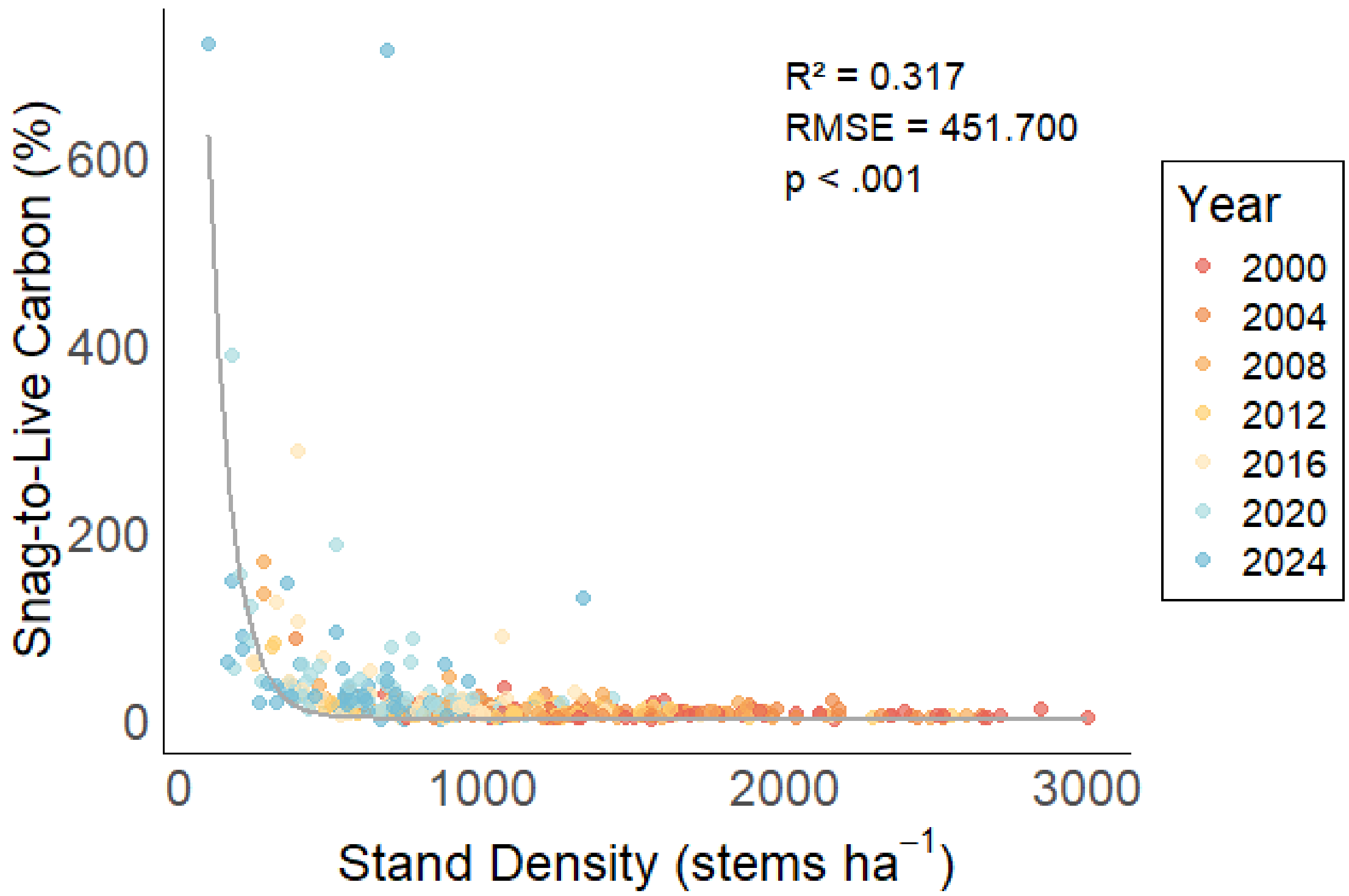

Figure S4. Relationships between the proportion of carbon stored in snags relative to live trees and stand density, for 64 stands sampled in the Climate Impacts on the Productivity and Health of Aspen project. Points represent stand-year observations and are coloured by survey year. A statistically significant negative relationship was found between stand density and snag-to-live carbon proportion ($p < .001$); the solid line represents the fitted general linear model with a log link function.

References

Huang, Shongming, Yuqing Yang, and Darren Aitkin. 2013. *Population and Plot-Specific Individual Tree Height-Diameter Models for Major Alberta Tree Species*. T/600. Alberta Environmental Protection, Land and Forest Services, Forest Management Division.

Lüdecke, D. 2025. *sjPlot: Data Visualization for Statistics in Social Science*. V. R package version 2.9.0. Released. https://CRAN.R-project.org/package=sjPlot.

Shaw, Cindy, Arlene Hilger, Michelle Filiatrault, and Werner Kurz. 2018. "A Canadian Upland Forest Soil Profile and Carbon Stocks Database." *Ecology* 99 (4): 989–989. https://doi.org/10.1002/ecy.2159.

Ung, Chhun-Huor, Pierre Bernier, and Xiao-Jing Guo. 2008. "Canadian National Biomass Equations: New Parameter Estimates That Include British Columbia Data." *Canadian Journal of Forest Research* 38 (5): 1123–32. https://doi.org/10.1139/X07-224.
